# Fully Immunocompetent CD8+ T Lymphocytes Are Present in Autologous Haematopoietic Stem Cell Transplantation Recipients Despite an Ineffectual T-Helper Response

**DOI:** 10.1371/journal.pone.0003616

**Published:** 2008-10-31

**Authors:** Alessandra Bandera, Daria Trabattoni, Michela Pacei, Francesca Fasano, Elisa Suardi, Miriam Cesari, Giulia Marchetti, Enrico M. Pogliani, Fabio Franzetti, Mario Clerici, Andrea Gori

**Affiliations:** 1 Division of Infectious Diseases, Department of Internal Medicine, “San Gerardo” Hospital, University of Milan-Bicocca, Monza, Italy; 2 Department of Preclinical Sciences, Chair of Immunology, LITA VIALBA, University of Milan, Milano, Italy; 3 Department of Clinical Sciences, Infectious Diseases Section, “Luigi Sacco” Hospital, University of Milan, Milan, Italy; 4 Clinic of Infectious Diseases, “San Paolo“ Hospital, University of Milan, Milan, Italy; 5 Hematology Division and Bone Marrow Transplantation Unit, San Gerardo Hospital, University of Milano-Bicocca, Monza, Italy; 6 Chair of Immunology, Department of Biomedical Sciences and Technologies, University of Milan and Don C. Gnocchi Foundation IRCCS, Milan, Italy; New York University School of Medicine, United States of America

## Abstract

**Background:**

Reduced CD4 T lymphocytes counts can be observed in HIV infection and in patients undergoing autologous haematopoietic stem cell transplantation (ASCT). Nevertheless, whereas opportunistic infections (OI) are frequent in HIV-infected individuals with low cell counts, OI are uncommon in ASCT patients.

**Methodology/Principal Findings:**

To verify whether this observation could be secondary to intrinsic HIV-correlated T cell defects, we performed in-depth immunologic analyses in 10 patients with comparable CD4 counts in whom lymphopenia was secondary either to HIV-infection or ASCT-associated immunosuppressive therapy and compared them to age-matched healthy subjects. Results showed the presence of profound alterations in CD4+ T lymphocytes in both groups of patients with respect to healthy controls. Thus, a low percentage of CCR7+ CD4+ T cells and a compensative expansion of CD45RA−CCR7− CD4+ T cells, a reduced IL-2/IFN-γ cytokine production and impaired recall antigens-specific proliferative responses were detected both in ASCT and HIV patients. In stark contrast, profound differences were detected in CD8+ T-cells between the two groups of patients. Thus, mature CD8+ T cell prevailed in ASCT patients in whom significantly lower CD45RA−CCR7− cells, higher CD45RA+CCR7− CD8+ cells, and an expansion of CCR7+CD8+ cells was detected; this resulted in higher IFN-γ +/TNFα production and granzyme CD8+ expression. The presence of strong CD8 T cells mediated immune responses justifies the more favorable clinical outcome of ASCT compared to HIV patients.

**Conclusion/Significance:**

These results indicate that CD8 T cells maturation and functions can be observed even in the face of a profound impairment of CD4+ T lymphocytes in ASCT but not in HIV patients. Primary HIV-associated CD8 defects or an imprinting by an intact CD4 T cell system in ASCT could justify these results.

## Introduction

High-dose chemotherapy followed by autologous stem cell transplantation (ASCT) has become an established treatment option for various malignancies [1], [2]. Although stem cell transplantation is believed to shorten the early period of severe neutropenia, recipients of ASCT suffer from a prolonged posttransplant immune deficiency, most pronounced in T-cell lineage, and cellular immunity remaining ineffective for several months after transplantation [3], [6]. Considering infectious complications, the early period of neutropenia is frequently complicated by bacterial episodes, but after neutrophils recovery, despite the persistent impairment of CD4+ cells and cellular specific immunity, the risk of developing opportunistic infections in ASCT patients is low [7]–[10]. In contrast to what is observed in ASCT recipients, the impairment in CD4+ T-cells in HIV infection, although in many cases less severe compared to ASCT, correlates with the risk of developing viral, fungal and bacterial infections, and a CD4+ count less of 200/µL is necessarily associated with major opportunisms development, AIDS-defining illnesses and death [11].

It follows that, since CD4+ T cells lymphopenia is not considered to be an univocal marker of risk for opportunistic infections, it is likely that various qualitative parameters of immune response play important roles. Interestingly, such a disease model, characterized by a discrepancy between T lymphocytes numbers and functions, was described early on in HIV research [12], [13].

A skewed maturation of HIV-specific CD4+ and CD8+ T-cells has been observed in HIV-infected patients, involving defective memory cell production and increasing defects in effector cell function, in conjunction with lack of antigen clearance [14]–[19]. Additionally, a selective loss of HIV-1 specific central memory CD4+ T cells, associated with an expanded monophenotypic CD4+ CD45RA−CCR7− response has been demonstrated, and there is strong evidence of the development of antigen-specific CD8+ T cells biased towards an effector, rather than a central memory phenotype. These phenotypic findings have been associated with the lack of control of the immune system on viral replication, owing to the fact that, in HIV infection, CD45RA−CCR7− CD4+ T cells are functionally characterized by low-IL-2/high interferon(IFN)-γ production and minimal proliferative ability, and CD45RA−CCR7− CD8+ T cells are endowed with lower content of lysis molecules, low proliferative ability, and altered cytokine production and ability [20]–[26].

Limited information is available regarding the phenotypic and functional heterogeneity of CD4+ and CD8+ T cells regenerating after high dose chemotherapy and ASCT; and to date no comprehensive characterization of maturative patterns has risen in these patients. The aim of our study was to compare phenotypic and functional patterns of T-cells in ASCT recipients and in HIV-infected subjects, with comparable CD4+ lymphopenia but with starkly contrasting infectious risks.

## Results

### Study groups

Ten age-matched subjects with comparable CD4+ lymphopenia were recruited: 5 cancer patients receiving ASCT 9 months before observation (ASCT) and 5 HIV+ antiretroviral *naïv*e subjects (HIV). Demographic and clinical data are presented in Table 1. According to institutional review board-approved chemotherapy protocols, high-dose chemotherapy for cancer patients consisted of ICE, ifosfamide (12 g/m^2^), carboplatin (1800 mg/m^2^) and etoposide (2 g/m^2^), for breast cancer patients (n = 2) and melphalan (cumulative dose 200 mg/m^2^) for multiple myeloma patients (n = 3). Febrile neutropenia was observed in 4/5 (80%) ASCT recipients during pre-engrafment phase and has been treated with large-spectrum empiric antibiotic. No opportunistic infections were observed in ASCT recipients until time of the analysis. Of HIV-infected patients, 4/5 had opportunistic infections during the six months previous to observation (2 patients esophageal candidiasis, 1 patient bacterial pneumonia and oropharingeal candidiasis and 1 patient thoracic VZV and oropharingeal candidiasis). Sulphamethoxazole/trimethoprim was administered as *Pneumocystis jiroveci* pneumonia prophylaxis to ASCT recipients and HIV-infected subjects with less than 200/µL CD4+ at observation. At time of the analysis, ASCT recipients and HIV-infected patients displayed comparable CD4+ absolute number and percentage: ASCT recipients 230/µl (range 164–300), 16% (range 10–25); HIV+ 255/µl (range 189–300), 17% (range 11–20) (p>0.05). CD8 lymphocytes absolute count and percentage did not show significant differences between the two groups: ASCT recipients 670/ml (range 496–919), 54% (range 48–69); HIV+ 658/ml (range 540–823), 51% (range 47–62) (p>0.05).

**Table 1:** Characteristics of the ASCT (Group A) and HIV-infected patients (Group B).

	Group A	Group B	p
	(n = 5)	(n = 5)	
**Inclusion criteria**			
CD4+ counts (cell/μL)	<300	<300	
Actual opportunistic infections	No	No	
Current HAART	Non applicable	No	
Neoplasia's relapse	No	Non applicable	
**Baseline characteristics**			
Age (years)			
median	48	43	ns
range	40–59	36–60	
Actual CD4+ T cells/mL			
median	255	230	ns
range	189–300	164–300	
Actual CD4+ %			
median	17	16	ns
range	11–20	10–25	
Actual CD8+ T cells/mL			
median	658	670	ns
range	540–823	496–919	
Actual CD8+ T %			
median	51	54	ns
range	47–62	48–69	
CD4/CD8 ratio			
median	0.34	0.29	ns
range	0.24–0.52	0.15–0.58	

Comparison of lymphocytes subsets phenotype and functional studies was made to data obtained from age-matched healthy subjects (n = 5) used as controls (HC), who displayed CD4+ and CD8+ absolute count and percentage within the normality range: CD4+ 1098/µl (range 797–2365), 45% (range 35.9–66.9); CD8+ 475 (range 447–628), 18.3% (range 17.7–21.3).

### CD4+ lymphocytes maturation patterns are skewed in ASCT recipients and in HIV-infected patients

**Figure 1:**
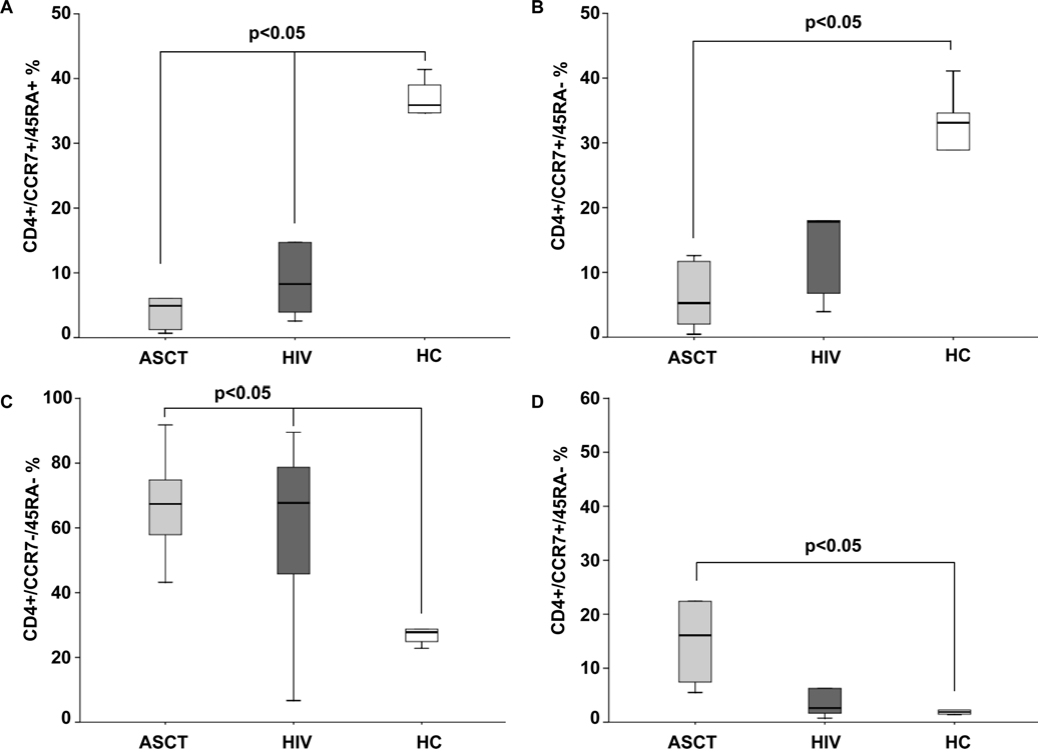
Phenotypical maturation pattern of CD4+ lymphocytes in ASCT recipients (ASCT), HIV-infected patients (HIV) and healthy controls (HC). The percentage of *naïve* CD4+ T cells (CD45RA+CCR7+) (A), *central memory* (CD45RA−CCR7+) (B), *effector memory* (CD45RA−CCR7−) (C) and *effector* cells (CD45RA+CCR7−) (D) were compared in the groups. ASCT recipients and HIV-infected patients displayed low percentages of *naïve* and central *memory* CD4+ T cells. A relative expansion of *effector memory* cells was observed in both population. Each central bar represents the median value and each box corresponds to the 25^th^ through 75^th^ percentile (interquartile) range.

We investigated phenotypical maturation pattern of lymphocytes in ASCT recipients and in HIV patients, analyzing cells based on the expression of CD45 isoforms and chemokine receptor CCR7. Despite contrasting clinical risk, ASCT recipients displayed a skewed CD4+ phenotype distribution very similar to what observed in HIV-infected subjects (Fig. 1). Indeed, the fraction of *naïve* CD4+ T cells (CD45RA+CCR7+) was significantly lower in both population compared to healthy controls (ASCT, 5.49%; HIV, 8.13%;HC, 35,9%; p<0.05 for HC versus ASCT and HIV; p>0.05 for ASCT versus HIV). Moreover, an expansion of *effector memory* CD45RA−CCR7− cells was seen both in ASCT and in HIV-infected patients with respect to age-matched healthy subjects (ASCT 62.6%; HIV 62.3%; HC 27.8%; p<0.05 for HC versus ASCT and HIV; p>0.05 for ASCT versus HIV), while ASCT recipients displayed the highest levels of *effector* CD45RA+CCR7− cells (ASCT 11.2%, HIV 2.18%, HC 1.89, p< 0.05 for ASCT versus HC, p>0.05 for ASCT versus HIV). *Central memory* cells (CD45RA−CCR7+), which have been recognized as the predominant CD4+ subpopulation in healthy controls together with *naïve* CD4+ T cells, were evidenced at lower frequency in ASCT recipients (8.48%) and HIV-infected subjects (17.9%), with statistical significance in the comparison between ASCT patients and healthy subjects (ASCT 8.48%, HIV 17.9%, HC 33.1%, p<0.05 for ASCT versus HC, p>0.05 for HIV versus ASCT and HC). Similarly to what observed in HIV-infected patients, ASCT recipients present a phenotypic CD4+ pattern characterized by a relative expansion of *effector* memory cells and a loss of *central memory* cells, which has been hypothesized disadvantageous in chronic infections.

### CD8+ maturation patterns are preserved in ASCT recipients compared to HIV-infected patients

**Figure 2:**
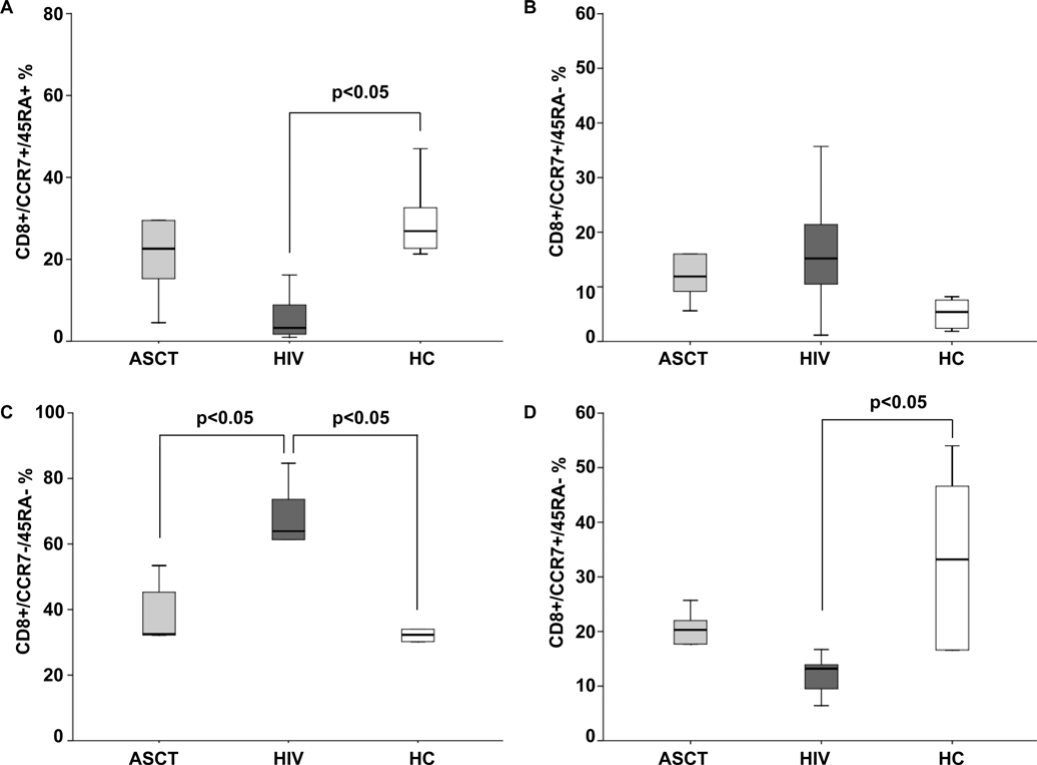
CD8+ T cell maturation patterns lymphocytes in ASCT recipients (ASCT), in HIV-infected patients (HIV) and healthy controls (HC). The percentage of *naïve* CD8+ T cells (CD45RA+CCR7+) (A), *central memory* (CD45RA−CCR7+) (B), *effector memory* (CD45RA−CCR7−) (C) and *effector* cells (CD45RA+CCR7−) (D) were compared in the groups. Higher levels of *naïve* CD8+ T-cells were observed in ASCT recipients compared to HIV+ patients. Whereas in HIV positive patients *effector memory* CD8+ T cells were the predominant memory phenotype, in ASCT recipients significantly higher levels of CD45RA+CCR7− *effector* CD8+ cells were evidenced. Each central bar represents the median value and each box corresponds to the 25^th^ through 75^th^ percentile (interquartile) range.

A wholly different scenario was evidenced when CD8+ T cell maturation patterns were analyzed in ASCT recipients and in HIV-infected subjects (Fig. 2). Indeed, while ASCT recipients displayed levels of CD45RA+CCR7+ CD8+ T-cells comparable with healthy controls (ASCT 28.83%; HIV 26.9%; p>0.05), significant reduced levels of *naïve* CD8 T cells were seen in HIV+ patients (6.07%, p<0.05 for HIV versus HC). Moreover, while CD45RA−CCR7− CD8+ T cells predominated in HIV positive patients, levels of these pre-terminally differentiated cells were significantly lower in ASCT recipients and in healthy controls (ASCT 36.4%, HIV 62.6%, HC 32.3%, p<0.05 for HIV versus ASCT and HC). Higher levels of CD45RA+CCR7− *effector* CD8+ cells were present in ASCT recipients than in HIV-infected subjects (ASCT 21.07%, HIV 12.55%, p = 0.05), who displayed significantly lower levels of terminal differentiated CD8+ T cells compared to healthy controls (HC 33.2%, p<0.05 for HIV versus HC). These data demonstrate that, unlike the chronic viral model, in ASCT recipients the rapidly replicating CD8+ T cell compartment follow a phenotypic distribution biased toward a terminally differentiated *effector* cell phenotype.

### A low effector memory/terminally differentiated CD8+ ratio characterizes ASCT recipients

To characterize the imbalance between T cells with different maturative phenotype, we calculated ratios of various subsets of CD4+ and CD8 lymphocytes across the two groups of patients. Ratios between CD4+ subpopulation, CD45RA−CCR7+/CD45RA−CCR7−, CD45RA−CCR7−/CD45RA+CCR7− and CD45RA−CCR7−/CD45RA+CCR7− evaluated for ASCT recipients failed to uncover significant differences compared to HIV-infected patients (0.12 versus 0.23, 6.83 versus 10.81, 0.57 versus 2.84 respectively). However, the comparison of ASCT recipients with age-matched healthy controls showed significantly low ratios between CM/EM, EM/TD, CM/TD CD4+ subpopulations in ASCT patients, thus confirming the skewed representation of repopulating memory CD4+ T cells.

**Figure 3:**
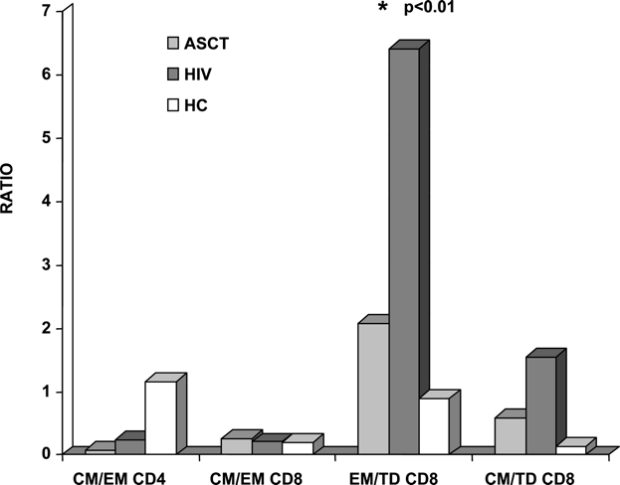
Ratios of CD4+ and CD8+ lymphocytes subsets across ASCT recipients (ASCT), HIV-infected patients (HIV) and healthy controls. ASCT recipients and HIV-infected patients displayed similar ratios between CD4+ subpopulations, CD45RA−CCR7+(CM)/CD45RA−CCR7− (EM). CD45RA−CCR7−CD8+(EM)/CD45RA+CCR7−CD8+(TD) ratio was significantly lower in ASCT recipients compared to HIV+ patients, with no differences in CD45RA−CCR7+ CD8+ (CM)/CD45RA−CCR7− CD8+ (EM) and CD45RA−CCR7+ CD8+ (CM)/ CD45RA+CCR7−CD8+ (TD) ratios among the two group of patients.

In stark contrast, the CD45RA−CCR7−CD8+ and CD45RA+CCR7−CD8+ ratio was significantly higher in HIV+ patients compared to ASCT recipients, who displayed values similar to healthy controls (ASCT 1.83, HIV 5.77, HC 0.97, p<0.05 for HIV versus ASCT and HC). This observation underlines the over-representation of pre-terminally differentiated CD8+ T cells and the simultaneously lower levels of terminally differentiated CD8+ T cells in HIV population (Fig. 3).

### Cytokine CD4+ production is impaired in both ASCT recipients and HIV-infected patients

**Figure 4:**
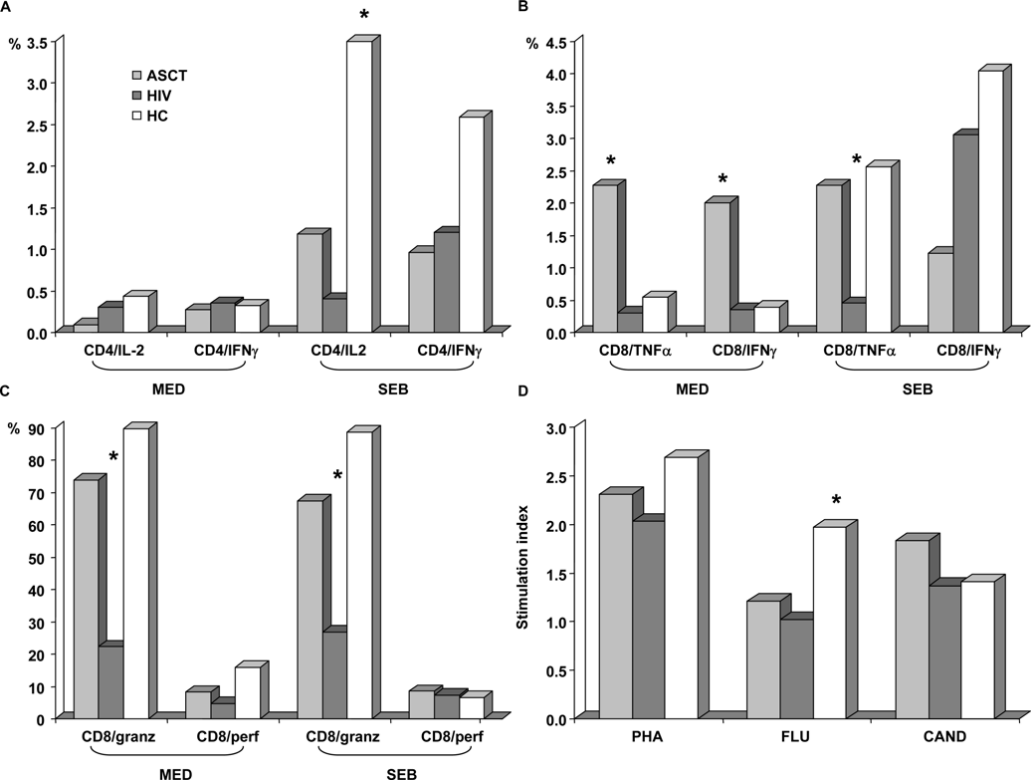
Cytokine CD4+ (A) and CD8+ (B) production, CD8+ lysis molecules expression (C) and lymphocyte proliferation (D) in ASCT recipients (ASCT), in HIV-infected patients (HIV) and in healthy controls (HC). ASCT recipients displayed significantly higher levels of TNF-α and IFN-γ producing CD8+ T cells under basal conditions and of TNF-α+ CD8+ T cells under *Staphylococcal* β enterotoxin (SEB) stimulation compared to HIV-infected patients. CD4+ IL-2 and IFN-γ production after SEB stimulation was globally depressed in both group of patients compared to healthy controls. The fraction of CD8+ T cells expressing granzyme B was higher in ASCT recipients than in HIV-infected patients. Antigen-induced proliferative responses showed comparable stimulation index values in both patients population and for polyclonal activation (PHA) as well as antigen stimulation (Flu, Candida), with significant difference with healthy controls regarding Flu stimulation. * = p<0.05 in the comparison between groups.

To determine whether the relevant phenotypic characteristics are associated with selective defects in cytokine production in ASCT recipients compared to HIV-infected subjects, we tested the capacity of CD4+ T cells to produce IL-2 and IFN-γ in response to superantigen stimulation (fig. 4A). Profound impairment in IL-2 and IFN-γ production after SEB stimulation was found in both group of patients with respect to age-matched healthy controls (IL-2+/CD4+ ASCT 1.18%, HIV 0.4%, HC 5.28%, p<0.05 for HC versus ASCT and HIV; IFN-γ+/CD4+ ASCT 0.96%, HIV 1.2%, HC 2.59%, p = ns).These observations suggest that CD4+ T cells activated via T-cell receptor stimulation are dramatically weakened in their capacity to produce IL-2 and IFN-γ, both in ASCT recipients and in HIV disease.

### Cytokine CD8+ production is observed in ASCT recipients but not in HIV-infected patients

In ASCT recipients, cytokine expression analysis for CD8+ T-cells produced a very different picture with respect to the HIV population (fig. 4B). Compared to HIV-infected patients, in fact, ASCT recipients showed significantly higher levels of TNF-α and IFN-γ producing CD8+ T cells (2.27% versus 0.3% and 2.00% versus 0.35% respectively, p<0.05). Moreover, differences were detected in cytokine production after SEB stimulation: CD8+ lymphocytes from ASCT recipients displayed significantly higher production of TNFα than those of HIV-infected patients (2.27% versus 0.45%, p<0.05) reaching levels similar to healthy controls (ASCT 2.27%, HIV 0.45%, HC 2.56%, p<0.05 for HIV versus ASCT and HC), despite comparable IFN-γ levels (ASCT 1.22%, HIV 3.05%, HC 4.04%, p = ns).

### Granzyme B expression

To better characterize the functional properties of cytotoxic cells, we additionally assessed by flow cytometry granzyme B expression in CD8+ T cells from ASCT recipients and HIV-infected subjects compared to healthy controls. Our results were consistent with those obtained using CD45RA/CCR7 co-expression, since the amounts of CD8+ T cells expressing granzyme B were many fold higher in ASCT recipients than in HIV-infected patients (ASCT 73.9%, HIV 22.3%, p<0.05) both in basal conditions and after SEB stimulation (ASCT 67.4%,HIV 26.8%, p<0.05) with no significant differences compared to age-matched healthy controls (basal 89.7%, after SEB stimulation 88.7%, p<0.05 for HC versus HIV, p>0.05 for HC versus ASCT)(fig. 4C). These findings correlate nicely with the phenotypic pattern described in ASCT recipients, considering that expression of granzyme B is a specific characteristic of mature effector CD8+ T cells.

### Lymphocyte proliferation

Finally, in order to investigate lymphocyte function, we analyzed lymphocyte proliferation in ASCT-recipients and in HIV-infected patients (fig. 4D). Antigen-induced proliferative responses were depressed in all patients with respect to age-matched healthy controls, reaching statistical significance for FLU stimulation (median stimulation index values: PHA ASCT 1.89, HIV 2.03, HC 2.69; FLU ASCT 1.55,HIV 1.02, HC 1.97, p<0.05 for HC versus HIV, *Candida albicans* ASCT 1.98,HIV 1.37, HC 1.40). One explanation for these observations could be that lymphoproliferation is primarily a function of T CD4+ cells, the numbers and functional capabilities of which were comparably depressed in both group of patients.

## Discussion

Delayed immune reconstitution remains one of the least understood issues among the many clinical challenges facing ASCT clinicians, and the clinical significance of profound and persistent CD4+ T cells lymphopenia, although often unassociated with opportunistic infections, has yet to be fully defined [27]. In patients with HIV infection, on the other hand, the selective loss of CD4+ T cells apparently triggers profound immunodeficiency, often leading to fatal infection, AIDS-defining illnesses and, ultimately, death [11]. To verify if the differing qualitative aspects of immune response characterizing CD4+-lymphopenic patients subjected to ASCT, with respect to those of HIV-infected patients, could account for the differences in infectious risks between the two groups, we phenotypically and functionally analyzed CD4+ and CD8+ lymphocytes in ASCT or HIV patients with comparable CD4 counts.

Our results demonstrate that patients undergoing high dose chemotherapy and ASCT have a persistent, marked deficit in CD4+ T-cell function. And yet, in contrast to HIV-infected subjects, such individuals are characterized by a CD8 T-cell compartment that is phenotipically normal and functionally active.

An alteration of the CD4/CD8 ratio, secondary to CD4+ T cells decrease and largely sustained by a loss of CD4+ *naïve* T cells, has often been described in different cohorts of ASCT recipients [3]–[5].

Our study confirms the profound loss of *naïve* CD4+ T cells in patients treated with high-dose chemotherapy and ASCT. Moreover, we show that the remaining CD4+ T compartment is constituted by T cells which have a *memory* phenotype, but which are skewed toward the later stages of differentiation, a characteristic typical of HIV infection. Central *memory* and effector CD4+ cells in fact were decreased both in HAART- *naïve* HIV-infected and in ASCT patients in whom a massive expansion of CD45RA−CCR7− effector *memory* CD4+ cells was observed. This phenotypic data well correlate with the results of our functional analysis, characterized by low IL-2/IFN-γ producing CD4 T lymphocytes of highly impaired proliferative ability.

In chronic viral infections, phenotypic and functional heterogeneity of memory CD4 T cells responses is believed to be regulated by antigen persistence and high/low levels of antigen load. The monophenotypic CD45RA−CCR7− CD4 T cell responses typical of chronic and progressive HIV infection have not, in fact, been found in long term non-progressors, where a multi-phenotypic CD4 T cell response, typical of repetitive Ag exposure and low Ag load was present [16], [23].

It is well known that lymphopenia is invariably associated with homeostatic expansion of the remaining T cells in the periphery, in response to endogenous MHC ligands and IL-7, but also with immune reactivity, both to self- and foreign antigens not normally apparent in T cell–replete hosts [28]. Consequentially, the explanation for the large compensative expansion of monophenotypic CD45RA−CCR7− CD4 T cell responses in ASCT recipients could be a high antigenic exposure to self peptide, including tumor antigens [29], or, alternatively, to nonself peptides, such as normal flora in the gastrointestinal [GI] tract–gut commensals, that have been recognized as the dominant force for rapid proliferation of T cells in severely immunodeficient host [28].

In summary, our data show that CD4+ T-cell count deficiency in ASCT recipients is associated with skewed representation of *memory* CD4 T cells and cytokine and proliferative defects - an immune pattern surprisingly similar to that of HIV infection. In contrast to HIV-positive patients, however, CD8 T cells were normally distributed and were functionally intact in ASCT recipients 9 months after. The presence of high quantities of TNFα/IFNγ-producing and granzyme-enriched effector CD8+ T cells, associated with the low rate of infectious mortality observed in ASCT recipients [8], suggest that even a T-cell compartment which is greatly impaired in CD4+ T cells number and function may provide protective immunity against opportunistic agents if it is enriched for effector antigen-specific CD8+ T cells. The protective effect of CD8+ T cells is supported by extensive clinical studies on HSCT patients. Analysis of immune reconstitution in both autologous or allogenic stem cell transplantation recipients showed that autologous recipients presented less viral reactivation associated with more vigouros CD8+-effector specific immune response [6]. Conversely, HIV-infected patients, as confirmed by our study, are characterized by the accumulation of pre-TD CTL, which phenomenon has been linked to the lack of control of the immune response on HIV replication in infected patients. It is therefore arguable that in a CD4+-lymphopenic setting, preservation of CD8+ effector function may be essential in the prevention of opportunistic infections.

Our observations indicate that although overall T CD4+ cell number and function are significantly reduced in ASCT recipients, the CD8+ T cell compartment is relatively well preserved. As animal models have elegantly shown, memory CD8 T cells generated in the presence of CD4 T cells gave the same robust secondary response after transfer into CD4-deficient or CD4-competent hosts [30]. Given the low rate of infectious mortality in ASCT patients, our data suggest that a CD8+ effector function, originally imprinted by a preserved CD4+ T cells system, might provide a clinically meaningful degree of protective immunity even in the absence of actual T-helper supports.

In conclusion, our study, even with limited data, confirms the substantial difference in both quantitative and functional regeneration of CD4+ and CD8+ cells after ASCT, providing strong evidence of a protective role played by differentiated T CD8+ cells in combating opportunistic infections and viral reactivation. We think that this issue together with the reasons of the skewed representation of memory CD4 T cells and cytokine and proliferative defects present in ASCT recipients should be explored more extensively in larger studies. In progressive HIV-infection, interventions aimed at expanding CD8+ T cell activity in the early phase of infection, when CD4 and CD8 T cell function is relatively preserved, may be an effective approach to improving global immunity and preventing opportunistic infections in advanced phase of immune-deficiency. Overall, these findings could prove critical in the clinical follow-up of ASCT recipients and for the design of vaccines in this population.

## Materials and Methods

### Study design

We performed a cross-sectional, observational, institutional review board-approved study of ASCT recipients and HIV-infected subjects attending the Chair of Infectious Diseases and Tropical Medicine–University of Milan, Italy.

Ten age-matched patients with comparable CD4+ lymphopenia (CD4+<300/µL) were enrolled according with the following definition: (i) neoplastic patients (breast cancer and multiple myeloma) (n = 5) receiving ASCT 9 months before observation, without neoplasia's relapse and free from opportunistic infections; (ii) HIV-infected patients (n = 5) *naïve* for antiretroviral therapy, stage B3 or C3 according to CDC ‘93 classification. Exclusion criteria were patients with recurrence of tumor or re-introduction of chemotherapy within 9 months after ASCT, patients with primary HIV infection defined as confirmed, recent HIV-1 infection by documented seroconversion illness and incomplete Western blot or a negative test within the previous 6 months, and previous treatment with cytokines or immunomodulants.

Comparison of lymphocyte subset phenotype and function was made to a data set obtained from 5 healthy controls aged 32 to 52 years (median 47 years) provided by the Chair of Infectious Disease and Tropical Medicine- University of Milan, Italy.

Patients and healthy controls participating in this study gave written informed consent according to the Declaration of Helsinki.

#### Flow cytometric analysis

Peripheral cells were washed in PBS and stained for 30 min at 4°C in the dark with the following antibodies: CD4 Cy5PE (mouse IgG2a isotype, Caltag); CD8 Cy5PE (mouse IgG2a isotype), CD8 FITC (mouse IgG2a isotype); CD45RA FITC (mouse IgG2b isotype); CCR7 PE (mouse IgG2a isotype, R&D Systems, Minneapolis, MN). Cells used for intracellular molecule staining were washed and fixed in Reagent A solution (FIX & PERM cell permabilization Kits; Caltag) for 10 min at room temperature in the dark. The cells were washed once again in PBS and resuspended in Reagent B (FIX & PERM cell permabilization Kits; Caltag) with cytokine- or perforin/granzyme-specific monoclonal antibodies (IL-2 PE; mouse IgG2a isotype; IFNγ FITC; mouse IgG1 isotype; TNF-α PE; mouse IgG2a isotype; granzyme B PE, mouse IgG1 isotype; Caltag Laboratories; perforin FITC; mouse IgG2b isotype; Ancell, Bayport, MN). The cells were then fixed in 1% paraformaldehyde in PBS.

Cytometric analyses were performed using an EPICS XL flow cytometer (Beckman-Coulter Inc., Miami, FL) equipped with a single 15 mW argon ion laser operating at 488 nm interfaced with 486 DX2 IBM computer (IBM, Cambridge, UK). For each analysis, 200,000 events were acquired. Green florescence from FITC (FL1) was collected through 525-nm bandpass filter and deep-red fluorescence from Cy5PE (FL4) was collected through 670-nm bandpass filter. Data were collected using linear amplifiers for forward and side scatter and logarithmic amplifiers for FL1 and FL4. Samples were first run using isotype controls or single fluorochrome-stained preparations for color compensation. The EPICS XL Coulter is 4-colour flow cytometer and a CD3 ECD mAb was used to gate T cells. Moreover, to be sure to identify only CD8+ T cells we analysed only CD8^bright^ cells that are CTLs, excluding CD8^dim^ cells which include NK cells.

#### Cytokine and perforin/granzyme production

PBMC were incubated for 18 hours with medium or *Staphylococcal* β enterotoxin (SEB) (5 µg/ml) (Sigma, St. Louis, MO). CD28 antibody (Clone 37407.111; R&D Systems, Minneapolis, Minnesota, USA) was added during incubation (1 µg/well) to facilitate co-stimulation. For cytokine and peforin/granzyme analyses 10 µg/ml Brefeldin A (Sigma, St. Louis, Missouri, USA) were added to the cell cultures during the last 6h of stimulation to block protein secretion. Analyses were performed on freshly collected cells. SEB concentration utilized in this study was tested to not induce cell death after overnight incubation. However, cells were analysed for vitality (blue trypan exclusion method) before ICC staining procedure.

#### Proliferative tests

PBMCs (3×10^5^) were placed in round-bottom wells of a microtiter culture plate (COSTAR, Cambridge, MA) in a final volume of 0,2 ml in the presence/absence of influenza virus vaccine prepared with a mixture of A/Taiwan, A/Shangai, and B/Victoria (FLU) (24 µg/ml); phytoemoagglutin (PHA) (2,5 µg/ml) (Sigma, St. Louis, MO); or *Candida Albicans* antigen (CAN) (1,6 µg/ml) (Green Laboratories, USA). Three replicate cultures were performed for each stimulation. Pooled human plasma (10%) were added to each well. We stimulated cells for 3 days to analyze lymphoproliferative responses to PHA and for 6 days to FLU vaccine prep and *Candida albicans*.

After incubation at 37°C and 7% CO2, cultures were pulsed with 1 µCi of [3H]-thymidine and harvested 18 h later. Incorporated [3H]thymidine was measured with a liquid scintillation beta counter. Results were expressed as mean counts per minute. The stimulation index represents the ratio of mean counts obtained in the presence of antigen to mean counts obtained without antigen.

### Statistical analysis

Procedures were based on non parametric analyses (Mann-Whitney); comparisons between the different groups were made using a two-tailed T-test. Statistical analysis was performed using the SPSS statistical package (SPSS Inc. Chicago, Illinois, USA).
